# Behavior of ligand binding assays with crowded surfaces: Molecular model of antigen capture by antibody-conjugated nanoparticles

**DOI:** 10.1371/journal.pone.0185518

**Published:** 2017-09-28

**Authors:** David C. Malaspina, Gabriel Longo, Igal Szleifer

**Affiliations:** 1 Biomedical Engineering Department, Northwestern University, Evanston, Illinois, United States of America; 2 Instituto de Investigaciones Fisicoquímicas, Teóricas y Aplicadas (INIFTA), UNLP, CONICET, La Plata, Argentina; 3 Chemistry Department and Chemistry of Life Processes Institute, Evanston, Illinois, United States of America; Brandeis University, UNITED STATES

## Abstract

Ligand-receptor binding is of utmost importance in several biologically related disciplines. Ligand binding assays (LBA) use the high specificity and high affinity of ligands to detect, target or measure a specific receptors. One particular example of ligand binding assays are Antibody conjugated Nanoparticles (AcNPs), edge-cutting technologies that are present in several novel biomedical approaches for imaging, detection and treatment of diseases. However, the nano-confinement in AcNPs and LBA nanostructures introduces extra complexity in the analysis of ligand-receptor equilibriums. Because antibodies are large voluminous ligands, the effective affinity in AcNPs is often determined by antibody orientation and surface coverage. Moreover, antibodies have two binding sites introducing an extra ligand-receptor binding equilibrium. As consequence of all this, experimental or theoretical studies providing a guidelines for the prediction of the binding behavior in AcNPs are scarce. In this work, we present a set of theoretical calculations to shed light into the complex binding behavior of AcNPs and its implications in biomedical applications. To investigate the ligand-receptor binding on AcNPs, we have used a molecular theory that predicts the probability of different molecular conformations of the system depending on the local environment. We have considered two different pathways for designing these devices: covalently conjugated antibodies and streptavidin-biotin conjugated antibodies. We also explore the effects of surface coverage, bulk concentrations, nanoparticle size and antibody-antigen affinity. Overall, this work offers a series of theoretical predictions that can be used as a guide in the design of antibody conjugated nanoparticles for different applications.

## Introduction

The binding between a ligand and its receptor is the main area of research in several biological related disciplines. Ligand-receptor binding is ubiquitous in many biological processes, including immune reactions, signaling, opening of ion channels and gene activity [1–4]. In the pharmaceutical industry, around 70% of the total sales of drugs to treat cancer [5] and autoimmune diseases [6] are therapies based on the binding of antibodies (the ligands) to specific receptors. The main feature of ligand-receptor binding that makes this interaction so attractive for a variety of applications is that it displays high specificity and high affinity. For example, antibodies only bind strongly to their respective complementary epitopes (high selectivity), with typical antibody-antigen dissociation constants (Kd) in the range of 10^−8^ to 10^−11^ M (high affinity) [7]. Due to these characteristics the biomedical research field has introduce several techniques that exploit ligand-receptor interactions. In particular, we can mention ligand-binding assays (LBA) that use ligands to detect, to target or to measure a specific receptor [1–3].

During the last decade, the production of LBA combined with nanoparticles (NPs) has increased due to the potential for in-vivo and in-vitro imaging and detection of different analytes, as well as for specific therapies such as thermal-ablation, gene therapy or localized drug delivery with nano-carriers [8–13]. However, nanoparticle mediated ligand-receptor binding displays a particularly complex behavior that arises from the confinement of the molecular species on a small surface. The chemical equilibrium between ligands and receptors can be locally displaced according to the inhomogeneous concentration of the species, which results in an effective affinity that highly depends on the local environment and the nature of the confinement. For that, predicting of the outcome behavior of LBA in nano-structures represents a challenging task that requires the collection of extensive experimental data and/or theoretical calculations.

The vast majority of novel LBA platforms include NPs modified with antibodies as ligands molecules. These antibody-conjugated nanoparticles (AcNPs) take advantage of the high affinity and selectivity of antibodies [5, 6, 14] in combination with the large surface-area/volume ratio offered by NPs. However, as mentioned before, confinement modifies the effective antibody-receptor affinity, which is highly dependent on the local environment. To add an extra layer of complexity, the effective affinity in AcNPs is often determined by the orientation and density of antibodies on the surface, since these molecules are large voluminous ligands. Moreover, antibodies have two possible binding sites, which introduces an additional ligand-receptor binding equilibrium.

Some experimental works on AcNP- and LBA-related systems have investigated the binding behavior as a function of antibody surface coverage [15–20]. These studies have shown that AcNPs ligand-receptor binding displays a non-monotonic behavior as a function of the number of antibodies on the surface. As the surface coverage of antibodies is increased, the amount of captured antigen reaches a maximum at low surface coverage and then decays into a plateau at high surface coverage. This non-monotonic behavior in the binding capacity is generally hypothesized as being the consequence of the increasing influence of surface crowding. Some theoretical works have focused on understanding the concentration dependence of this binding [21], but they have not conclusively explained the non-monotonic behavior in AcNPs.

Ligand-receptor binding in LBA and AcNPs is favored by the decrease in chemical free energy, particularly when the affinity is high. In contrast, on crowded surfaces with voluminous ligands, several entropic contributions to the free energy oppose this binding (*e*.*g*., the entropic loss due to ligand immobilization). This decrease in entropy upon binding is particularly relevant if the nano-environment near the surface is sufficiently crowded. In this work, we will show that ligand-receptor binding with voluminous molecules such as antibodies results from the competition between binding and opposing entropic effects induced by surface crowding.

Our approach to study this problem is to use a molecular theory based on the minimization of the total free energy of the system. This molecular theory [22–26] take into account the conformations and chemical information of each of the molecules involved, including a detailed description of the antibody three dimensional structure. As a result, we can calculate the thermodynamic properties of the system, the density profiles and the degree of binding of the different species on or near the surface. Previous works have used this theory to investigate the behavior of ligand-receptor binding in tethered polymer layers [23, 24] showing good agreement with a similar experimental findings [20, 27] Moreover, this theory has shown very good agreement with experimental results in different scenarios[22–26, 28].

**Fig 1:**
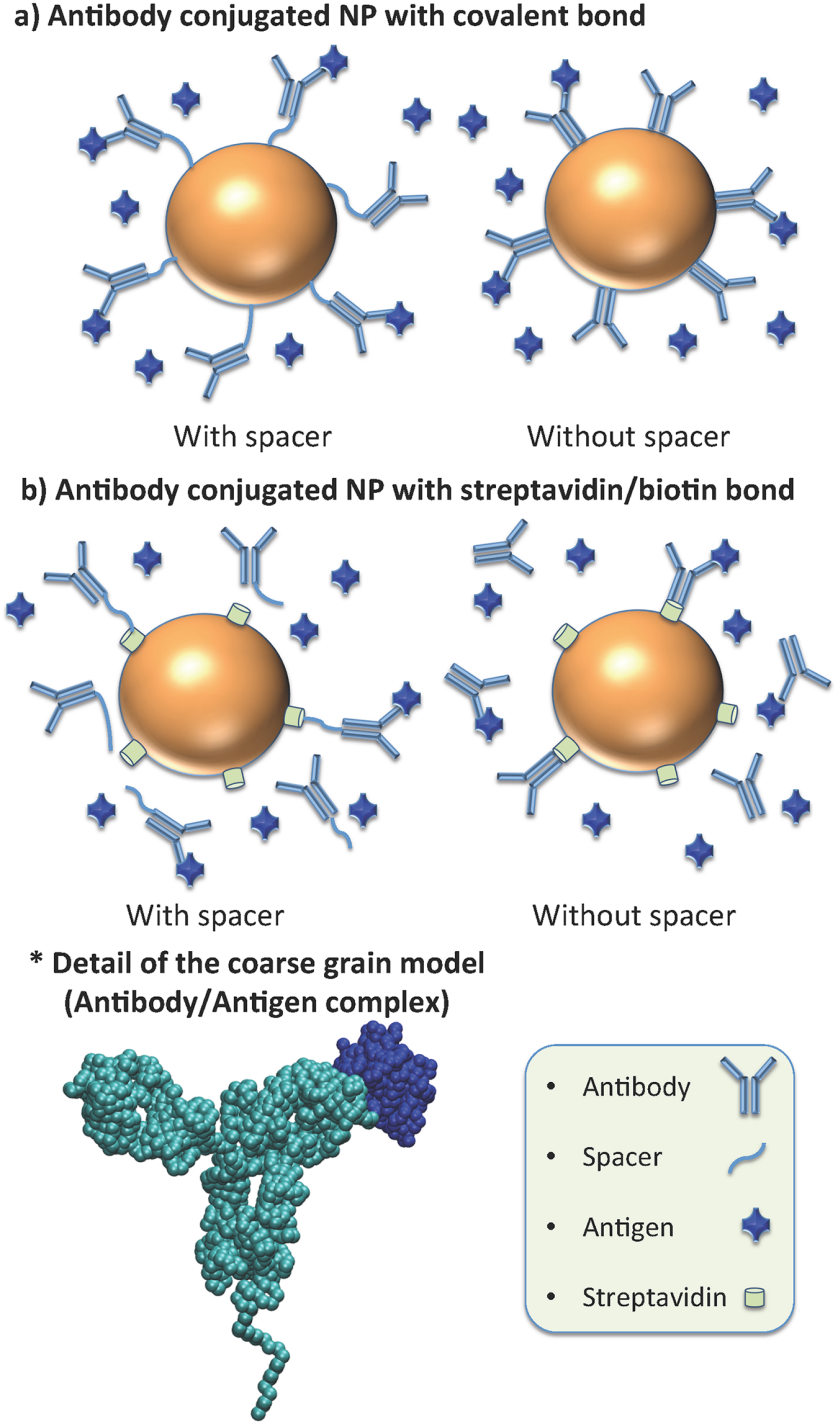
Scheme of the systems investigated in this work. a) Antibody conjugated nanoparticle with antibodies covalently bonded to the nanoparticle. b) Antibody conjugated nanoparticle with antibodies bonded through streptavidin-biotin. *) Scheme of the coarse grain model of the antibody b12 (cyan) having a 50 units spacer and an antigen gp120 bound (blue).

Here we use this molecular theory to investigate ligand-receptor binding of LBA and in particular of AcNPs. We study the molecular origin of the non-monotonic binding behavior in AcNPs and predict the optimal surface coverage for different system configurations as a function of several variables. Moreover, there are different conjugation strategies to anchor antibodies to AcNPs [29], some relying on a second ligand-receptor equilibrium with high affinity (*i*.*e*., biotinylated antibodies that bind to streptavidin on the nano-surface). Though strong, this second binding is not permanent. To investigate this possibility, we have evaluated two different designs: a) AcNPs with antibodies covalently grafted to the surface, and b) AcNPs with antibodies conjugated through streptavidin-biotin binding. Fig 1 schematizes the different systems considered in the present work, as well as the coarse-grained molecular model used to calculate results with our theory. More details about the theoretical approach can be found in method section and in the supporting information (S1 Text).

In the following sections we will present and discuss the results obtained in our calculations and show the dramatic effect that voluminous ligands have in determining ligand-receptor equilibrium in confined environments. Furthermore, the following set of results provides a theoretical platform for facilitating the interpretation of experimental findings in similar complex scenarios and can help in the rational design of biomedical applications of LBA and AcNPs.

## Methods

Our theoretical methodology is based on a previously developed molecular theory [15–20]. The predictions of this theory have been shown to be in good agreement with experimental observations for ligand-receptor binding in polymer layers [20, 23, 24, 27]. This method explicitly accounts for the volume and conformations of the molecular species including the antibody, antigen and antibody-antigen complexes, and predicts the amount of binding depending on local environment. Application of this theory requires the input of the different molecular conformations of the system. To describe the antibody, we use a coarse-grained model based on the crystallographic data of anti-HIV-1 b12 monoclonal antibody [30] and its complex with HIV gp120 [31]. For the binding of two antigens to the antibody we have used a ratio affinity/avidity extracted from experimental results on b12 antibody [32]. The dissociation constant range is based on the experimental work of Landry et al [7] that suggest a Kd range of 10^−8^ to 10^−11^ M for different antibody/antigens. Each amino acid in the structure is represented by a single bead of 0.6 nm diameter, centered at the alpha carbon of the corresponding residue. The spacer conformation is based on a coarse-grain model of poly(ethyleneglycol) (PEG) where each bead (diameter = 0.6 nm) represents a monomeric unit (EG). The antibody structure is fixed in its initial conformation obtained from the PDB, but has full three-dimensional rotational and translational freedom. Conformations of the spacer are generated using a rotational isomeric state model [33]. Streptavidin is considered as punctual binding sites on the surface having no volume.

The general analytical form of the Helmholtz free energy used to describe this system is the following:

$$\begin{array}{c} \beta F=4\pi \int_{R_{NP}}^{\infty }dr\;r^{2}\;\rho_{w}\left(r\right)\left[ln\;\rho_{w}\left(r\right)v_{w}-1\right]+\sum_{j\in \left\{a,A,Aa,Aaa\right\}}\left(4\pi \int_{R_{NP}}^{\infty }dr\;r^{2}\;\sum_{\alpha_{j}^{sol}}\rho_{j}\left(\alpha_{j}^{sol},r\right)\left[ln\;\rho_{j}\left(\alpha_{j}^{sol},r\right)v_{w}-1+\beta \mu_{j}^{⊖}\right]\right)\\ +N_{S}\sum_{i\in \left\{A,Aa,Aaa\right\}}f_{i}^{NP}\left(\sum_{\alpha_{i}^{NP}}P_{i}\left(\alpha_{i}^{NP}\right)ln\;P_{i}\left(\alpha_{i}^{NP}\right)\right)+N_{S}\sum_{k\in \left\{S,A,Aa,Aaa\right\}}f_{k}^{NP}\left(ln\;f_{k}^{NP}+\beta \mu_{k}^{NP⊖}\right) \end{array}$$

The different terms in this equation account for: the translational entropy of water (first term), the translational and conformational (rotational) entropy of the species in solution as well as their self-energies (second term), the conformational entropy of those species bound to the surface (third term), and the mixing entropy and self-energy of the species bound to the surface (fourth term). The subscripts represent the different species: unbound antibody (A), antibody + 1 antigen (Aa), antibody + 2 antigens (Aaa), streptavidin (S) and water (w). ρ_i_(r) is the local density of species “i” in the element of volume of thickness dr at a distance r from the center of the spherical nanoparticle. The volume of a water molecule is *v*_*w*_. The different molecular conformations of a species are denoted by α_i_^NP^ for molecules bound to the nanoparticle surface and α_i_^sol^ for free molecules in the solution. *P*_*i*_(α_i_^NP^) is the probability of having a molecule of species “i” in a conformation “α_i_^NP^” attached to the NP. N_s_ is the number of active sites on surface (streptavidin or antibody). *f*_i_^NP^ is the fraction of active surface sites occupied by the specie “i”. The quantity μ_i_^NP0^ and μ_i_^NP0^ are the standard chemical potentials of the bound and free specie “i”, respectively, and β = 1/k_B_T where k_B_ is the Boltzmann constant and T the temperature. In this description there are no explicit interactions included and all the attractions are grouped in terms of dissociation constants Kd. The repulsive part of the interaction is taken into account as the excluded volume occupied by different species.

Functional minimization of the free energy yields expressions for the each of above-described functions that depend on one interaction potential, the local osmotic pressure. This minimum must satisfy local incompressibility of the fluid and constant chemical potential of the species in solution. To solve this constrained optimization, we introduce Lagrange multipliers. The local osmotic pressure is obtained by numerically solving the incompressibility constraint at each distance (in the solution) from the center of the nanoparticle. Calculations are performed in radial coordinates (r), and space is discretized in layers of thickness dr. Once we obtain the interaction potential, all the functions that compose the free energy are determined. Then we can calculate the number antigen/antibodies adsorbed as a function of the number of binding sites on the surface (or the number of antibodies grafted to the surface). Moreover, any thermodynamic quantities of interest can be derived from the free energy. Structural properties can be calculated using the probability distribution of the different molecular conformers. More specific details of the theory and mathematical procedure employed are provided in the supporting information (S1 Text).

Our calculations are performed at constant chemical potential (constant concentration). Thus, in order to investigate the degree binding, we have to include the bulk species of all the possible complexes. However, in the covalently conjugated and streptavidin conjugated AcNPs, we have different amounts of possible complexes. Nevertheless, in order to compare the binding behavior in the two AcNP architectures, some results are plotted as a function of the number of bound antibody-antigen pairs on surface. Moreover, in the streptavidin conjugation scheme, the equilibrium concentration of each species in solution is obtained through solving the chemical equilibrium for the initial conditions. These initial conditions yield similar concentrations as those considered for the system with covalently bonded antibodies.

## Results

### a) AcNP with antibodies covalently bonded

Our first ligand-receptor system consists of a spherical NP with a fix number of antibodies (ligands) attached to the surface immersed in an antigen (receptor) solution of controlled concentration. Antibodies are grafted to the surface with a chemical bond. The variables that we control on the theoretical model are: spacer, antibody/antigen dissociation constant (Kd), bulk concentration and antibody surface coverage. To analyze relevant concentrations of antigen, we consider two different experimental situations. Since we are using the molecular architecture of anti-HIV-1 antibody, we use an antigen (gp120) bulk concentration of 20 pM, in range with the observed concentrations in serum at the acute stage of HIV infection [34, 35]. On the other hand, to illustrate a more concentrated environment like *in vitro* systems we have used a concentration of 100 nM of antigen. The NP radius used in these calculations is 50 nm, which is in the typical range for polymeric conjugated nanoparticles.

**Fig 2:**
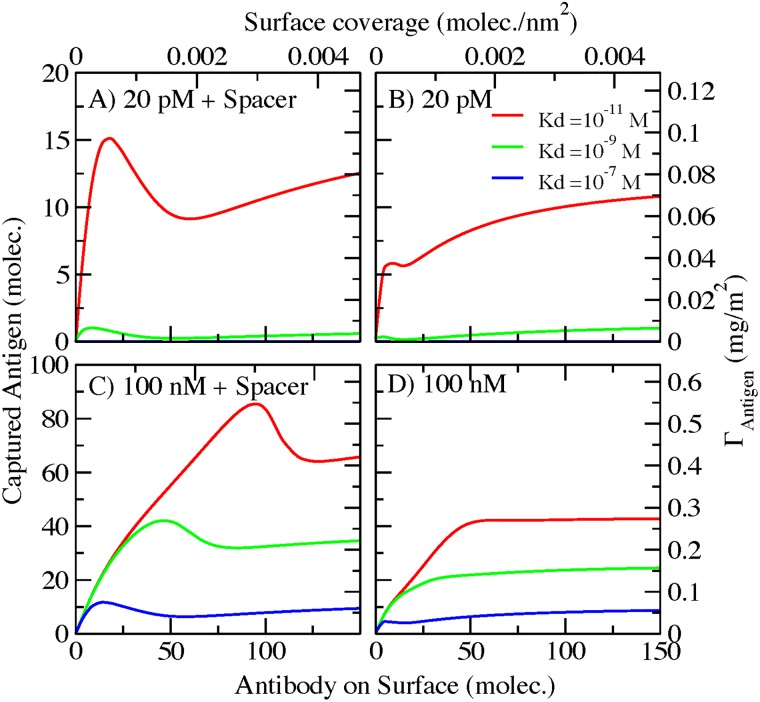
Captured antigen as a function of antibody surface coverage on AcNP with antibodies covalently conjugated. The different colors represent different antibody/antigen Kd values. A) Antigen bulk concentration = 20 pM and spacer of 50 monomers. B) Antigen bulk concentration = 20 pM and no spacer. C) Antigen bulk concentration = 100 nM and spacer of 50 monomers. D) Antigen bulk concentration = 100 nM and no spacer.

In Fig 2, we plot the number of antigens captured by the AcNP as a function of the surface coverage of antibodies, for an antigen bulk solution of 20 pM (upper panels, Fig 2A and 2B) and 100 nM (lower panels, Fig 2C and 2D) respectively. This functionality has been evaluated for two spacer lengths: 50 segments (left panels, Fig 2A and 2C) and no spacer (right panels, Fig 2B and 2D), and for 3 different antibody/antigen dissociation constants (different color lines). System behavior is completely different in all the studied possibilities (Fig 2 panels A to D). This is an indication of the complexity of ligand-receptor equilibrium at the surface of the AcNPs. At low antigen bulk concentration (Fig 2A and 2B; 20 pM antigen), only antibodies with a very high affinity (Kd = 10^-11^M) can capture antigen molecules. Antigen-antibody pairs with such strong affinity are not frequent in nature [7], which implies that a wide range of applications, these AcNPs will not bind to any receptors at this concentration. Fig 2B displays the behavior at low antigen bulk concentration without a spacer, and Fig 2A does with the addition of a spacer of 50 segments. In Fig 2A shows the presence of a maximum at low surface coverage. This maximum is the result of the competition between ligand-receptor binding and conformational entropy of species on the surface. As the number of antibodies on the surface of the AcNP increases, as many as possible ligand-receptor pairs bind. The maximum is reached when adding more antibodies on surface significantly reduces the conformational possibilities of the system. If we continue adding more antibodies to the surface, the number of bound ligand-receptor pairs decreases to an approximately constant value (plateau), showing the point where adding more antibodies does not increase the amount of bounds pairs on the AcNP.

Introducing a spacer slightly increase the amount of bound pairs on the AcNP (Fig 2A and 2B). The function of the spacer is to increase the conformational possibilities of the antibodies on the surface. This conformational gain is due to the flexibility of the spacer leading to an increment in the available volume for binding, which can occur at different distances from the surface. Due to this entropic effect, the spacer (Fig 2A) enhances the presence of the maximum observed at low surface coverage.

The addition of a spacer considerably increases the amount of bound pairs on the AcNP (compare Fig 2C and 2D). As we have previously noted in Fig 2A, spacer presence leads to a maximum in the amount of bound pairs at low surface coverage. The maxima in Fig 2C are also shifted to higher surface coverage as we increase the antigen-antibody affinity. These two features are the result of the further displacement of the ligand-receptor equilibrium conditions towards the bound state that occurs nears the surface when increasing the receptor bulk concentration.

**Fig 3:**
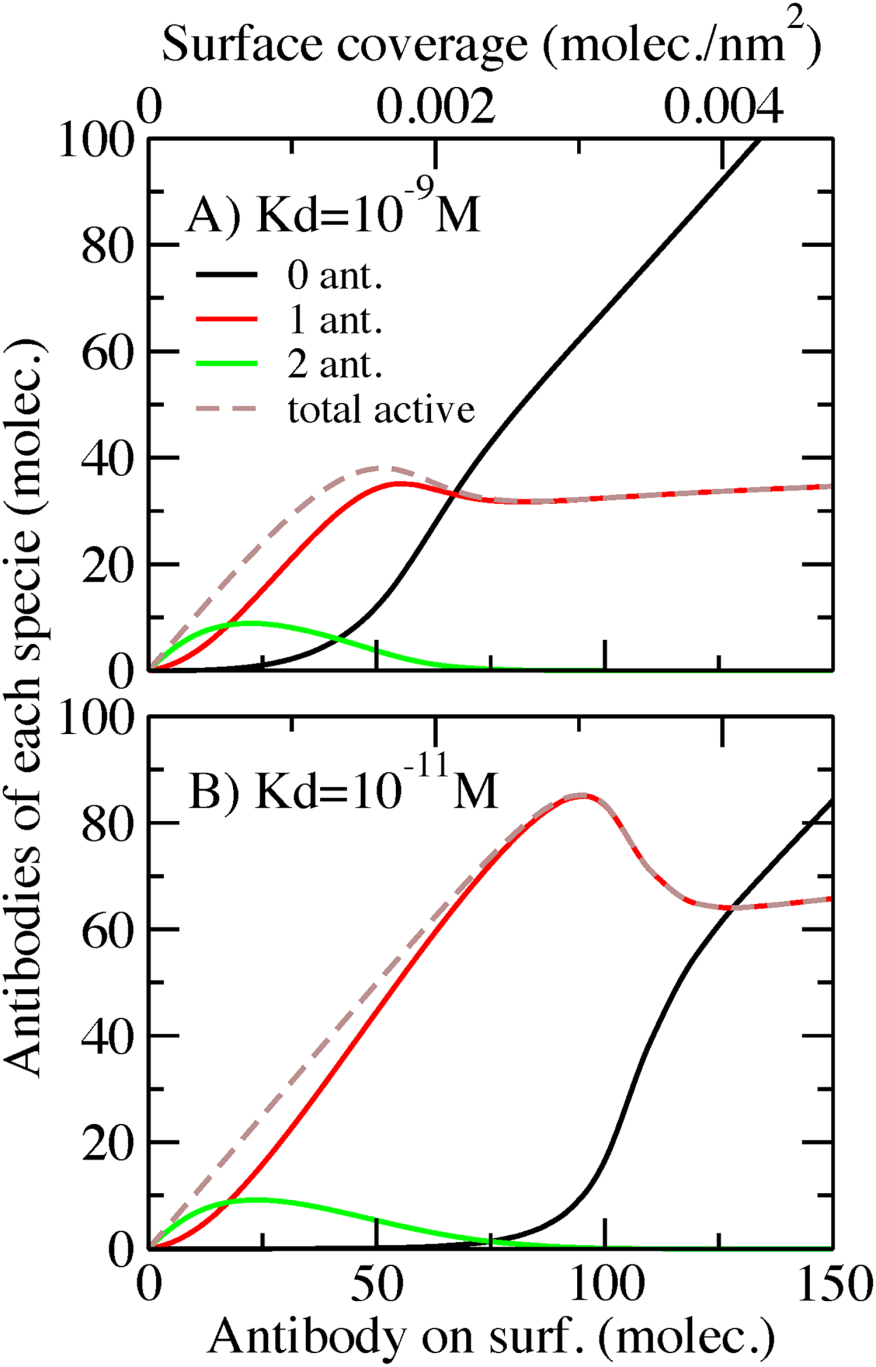
Number of unbound antibodies (0 ant.), antibodies bound to one antigen (1 ant.), antibodies bound to two antigens (2 ant.) and total active antibodies (total) on surface, as a function of the surface coverage in a covalently bonded AcNP. A) Antigen bulk concentration = 100 nM, 50 monomer spacer, Kd = 10^−9^ M. B) Antigen bulk concentration = 100 nM, 50 monomer spacer, Kd = 10^−11^ M.

One of the advantages of our molecular theory is that we can obtain information about the amount of each species on the surface: unbound antibody, antibody bound to one antigen and antibody bound to two antigens (one in each Fab domain). We are considering only monoclonal antibodies; then the possible binding events are one antigen in each Fab domain (two ligand-receptor equilibriums). In Fig 3, we plot the contribution of each species and the total number of active antibodies on surface at high antigen bulk concentration (100 nM) and a 50 monomer spacer for Kd = 10^−9^ M (Fig 3A) and Kd = 10^−11^ M (Fig 3B). Fig 3 represent the same conditions as the red and green curves of Fig 2C. The dashed straight lines represent the total number of antibodies bound to antigens. In Fig 3A (Kd = 10^−9^ M) we observe that antibodies with two antigens are only present at low surface coverage (Fig 3 green lines). These are the complexes that occupy the largest volume. When surface crowding increases, antibodies with two antigens become the least favorable species due to the high entropic cost of crowding. The species predominantly contributing to the antigen capture capability as shown in Fig 3A is the antibodies with one antigen attach to the fab domain (red line curve). The plot also shows that the number of antibodies with one antigen grows almost linearly with surface coverage until reaching the conditions where crowding starts to affect binding. Maximum binding occurs at approximately 0.00159 antibodies/nm^2^. After reaching this maximum, the amount of antibodies with one antigen shows a plateau. At the same time, as we increase the surface coverage we are only adding to the surface antibodies that will remain unbound (Fig 3 black line).

A similar behavior can be observed in Fig 3B at Kd = 10^−11^ M, but the shape and position of the maxima are shifted to higher surface coverage. The maximum for antibodies with one antigen is displayed at approximately 0.003 antibodies/nm^2^. As mentioned before, a lower Kd leads to a more pronounced non-monotonic effect. Interestingly, Fig 3A and 3B show similar populations of antibodies with two antigens. This indicates that despite the gain in chemical free energy, the entropic cost of having two antigens attached to an antibody is very high, and not even reducing the Kd (increment in affinity) two orders of magnitude substantially enhances the presence of this molecular complex.

**Fig 4:**
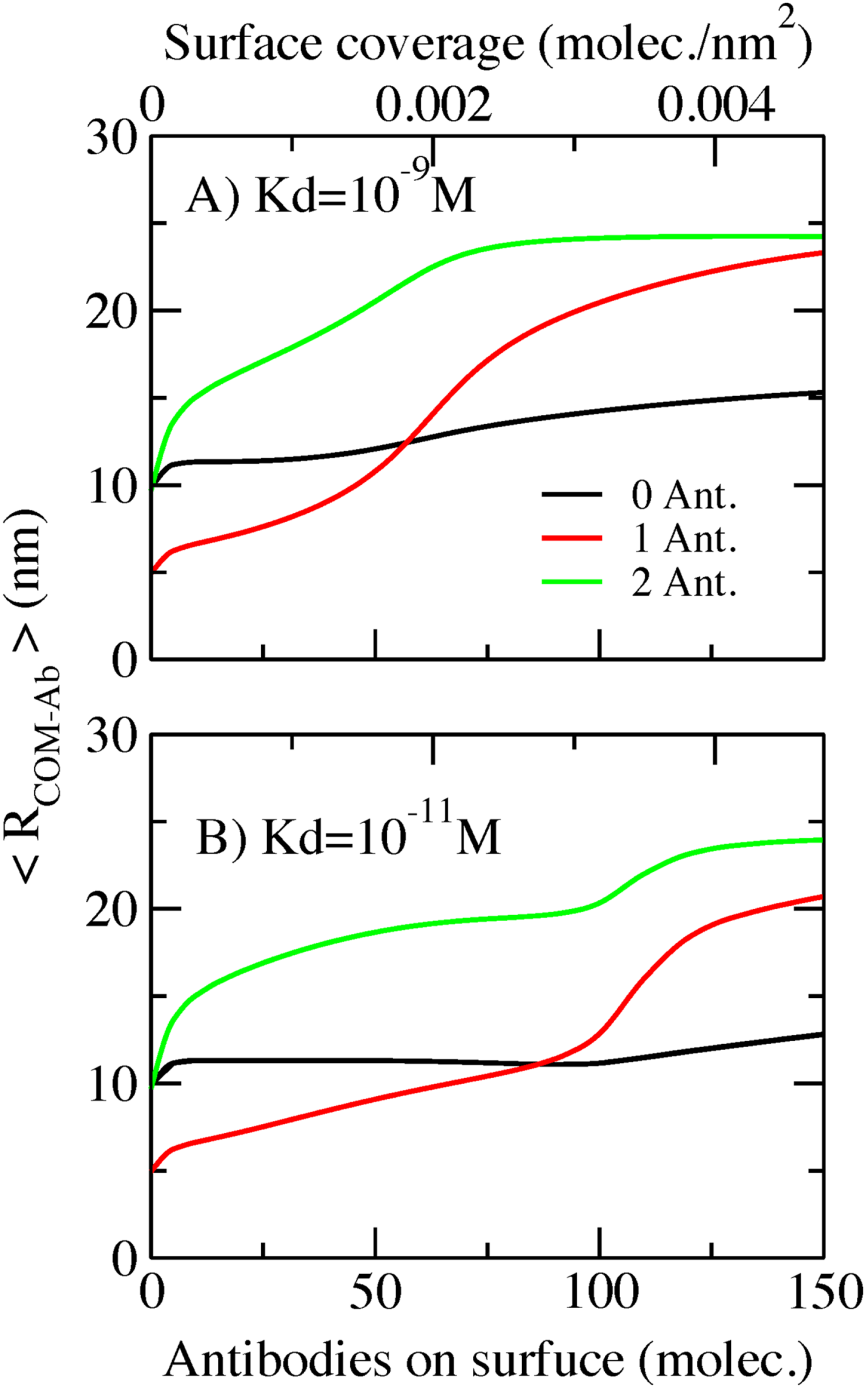
Covalent bonded AcNP. Averaged center of mass of unbound antibodies (0 ant.), antibodies bound to one antigen (1 ant.), antibodies bound to two antigens (2 ant.) as a function of the surface coverage. A) Antigen bulk concentration = 100 nM, spacer 50 monomers, Kd = 10^−9^ M. B) Antigen bulk concentration = 100 nM, spacer 50 monomers, Kd = 10^−11^ M.

Our theoretical approach offers the possibility of analyzing the probability of the different molecular conformations. Using such information allows for calculating different quantities to describe molecular reorganization on the surface of the AcNP. Fig 4 shows the average value of the position of the center of mass of the antibody (<R_COM_>). The conditions are the same as those of Fig 3, bulk antigen concentration of 100 nM and a spacer having 50 monomeric units. In Fig 4A, Kd = 10-9M and Kd = 10-11M in Fig 4B. The center of mass position gives information about how stretched or compacted are the molecules on the surface as a function of surface coverage. Higher values of <R_COM_> represent antibodies far from the surface in more stretched conformations of the spacer. We observe in Fig 4A that antibodies bound to two antigens (green line) are the ones that remain farthest from the surface. As aforementioned, this complex occupies the largest volume. Then the optimal conformations of this species are stretched because more compact conformations would lead to high steric repulsions. Unbound antibodies (black line), in contrast, remain in relatively collapsed conformations with little variation in the position of their center of mass.

The conformations of antibodies with one antigen (red line curve in Fig 4A), remain relatively collapsed at low surface coverage, while at high surface coverage they are found in more stretched conformations due to crowding effects. Interestingly, both antigen-antibody complexes, with one and two antigens, undergo a transition from compact to stretched conformations around the positions (surface coverage) of the their respective maximal binding (see Fig 3A). This implies that those maxima are reached when crowding induces the transition from collapsed to stretched conformations. Looking at Fig 3A, we see that antibodies with two antigens vanish when more than ~75 antibodies are on surface, which is due to the high entropic loss of having fully stretched conformations. The features of the above described conformational behavior of surface antibodies are similar for different binding affinities, the difference being the position of the transition from collapsed to stretched conformations. In Fig 2C, we show that a stronger binding affinity results in the displacement of maximum binding towards higher antibody surface coverage. This occurs because the decrease in chemical free energy upon binding is higher for antibodies for antigens with stronger affinities, which decreases the relative entropic penalty of surface reorganization. For stronger binding affinities, the transition from compacted-to-stretched conformations occurs at higher surface coverage, thus shifting the position of the maximum binding capacity of the AcNP to higher surface coverage as well.

**Fig 5:**
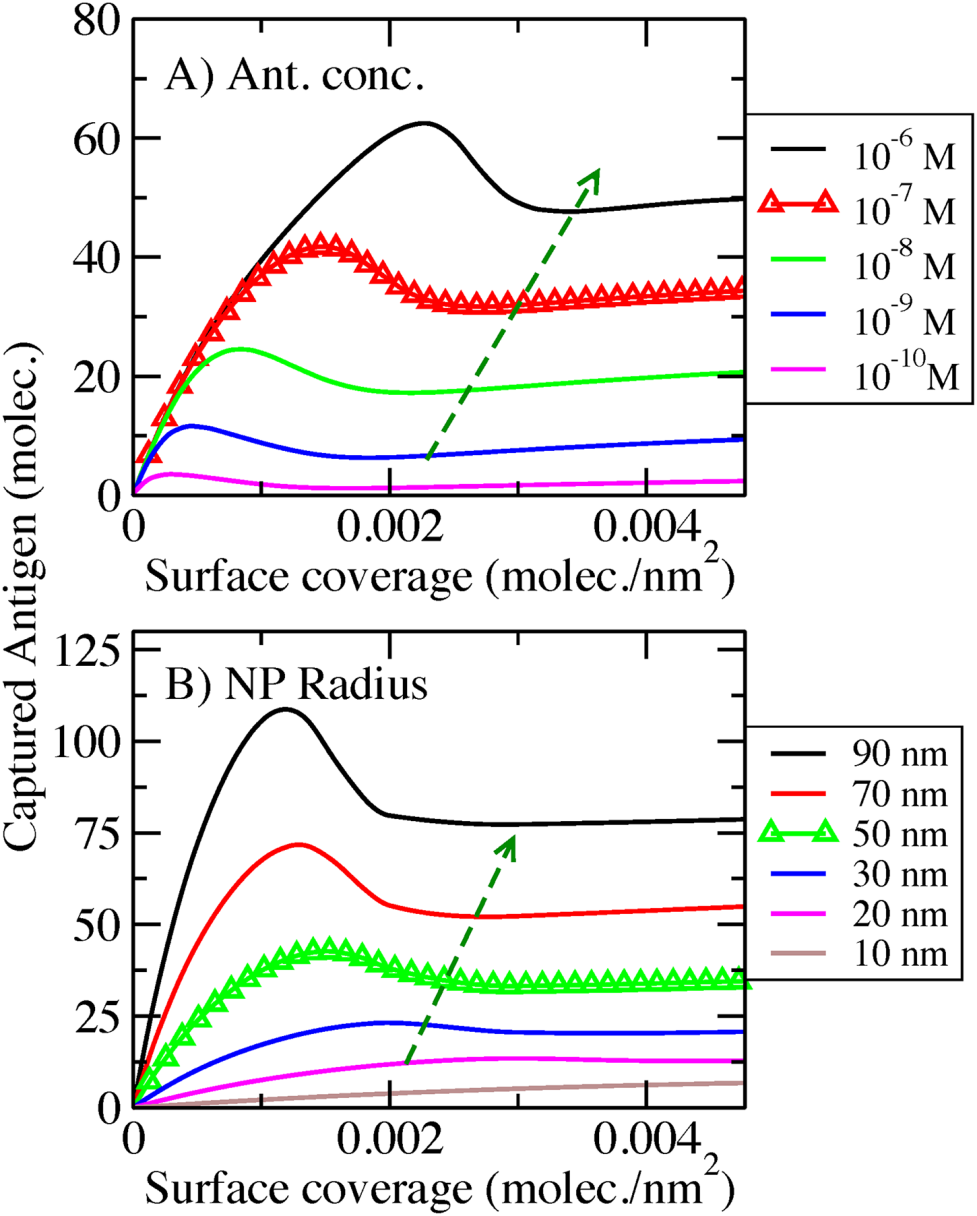
Captured antigen per area as a function of antibody surface coverage. Covalent bonded AcNP, spacer 50 monomers, Kd = 10^−9^ M. A) For different antigen bulk concentrations. AcNP radius = 50 nm. B) For different AcNP radius. Inset in panel B) are same data but the captured antigen normalized by the area of the NP. Red and black dashed lines, in the inset, represent the ideal situation of all the antibodies bound to two or one antigen respectively. Antigen bulk concentration = 100 nM. Triangle symbols represent the set of conditions used in this paper for the rest of the calculations.

We next analyze the role of bulk concentration of antigen and nanoparticle radius in the binding of antigens. Fig 5 presents results for an AcNP with covalently conjugated antibodies, with a spacer of 50 monomers and Kd = 10^-9^M, for different bulk concentrations of antigen (Fig 5A) and different NP radius (Fig 5B). In this plot, dashed arrows indicate the direction in which either the concentration or the radius is increased.

Fig 5A shows the influence of bulk concentration of antigen on the ligand-receptor binding. As we increase this bulk concentration, the non-monotonic binding behavior (maximum) enhances. This effect is similar to what occurs upon increasing the antibody-antigen affinity (Fig 2C). A higher bulk concentration displaces chemical equilibrium to favor bound pairs. Namely, raising the bulk concentration increases the chemical potential of the solution favoring binding. However, at a given surface coverage crowding effects start to play an important role (maximum binding). After this condition, many conformations that do not optimize volume become unfavorable (binding displays a plateau).

Fig 5B shows the dependence of the amount of bound pairs on the nanoparticle radius. AcNP curvature strongly influences the non-monotonic binding behavior. This response can be interpreted in terms of crowding. For large AcNP radius, the surface is locally planar, which limits the conformational possibilities. In contrast, for smaller AcNP radius, the high curvature introduces additional volume, which allows for more conformational freedom. As we see in Fig 5B, the maximum progressively vanishes with decreasing NP radius, indicating the weaker crowding effects at higher curvatures (lower radius). This phenomenon is particularly prominent for an AcNP of 10nm radius, where the binding behavior becomes almost linear with increasing surface coverage.

In Fig 5B we also included an inset with the same data but the number of captured antigen had been normalized by the area of the nanoparticle. This inset provide information about the efficiency of binding. Red and black dashed lines in the inset represent the ideal situation of all the antibodies bound to two or one antigen respectively. We can observe that AcNP with smaller diameters tend to be more efficient in capturing antigen. As we mentioned before, this is a consequence of the increment in curvature that provides more available volume and then less entropic penalty to the binding.

### b) Antibody conjugated NP having antibodies bound through biotin/streptavidin

We have also investigated a system consisting of an AcNP with antibodies conjugated through a streptavidin/biotin bond. This system is particularly interesting due to its many biomedical applications [8]. In spite of the strength of the streptavidin-biotin binding (Kd~10^−14^ M), this ligand-receptor equilibrium has a quite different behavior on nano-scale surfaces [25]. This fact is oftentimes overlooked in experimental design considerations where the strong bond is frequently assumed to be permanent. Then, we address the main differences between the previously studied system (an AcNP with covalently bound antibodies) and an AcNP with streptavidin-biotin bound antibodies. We consider similar experimental conditions, but we should note that this system have more species in solution due to an additional ligand-receptor equilibrium (biotin-streptavidin). The equilibrium concentrations in solution for each species are calculated based on the initial concentration of antibody and antigen.

**Fig 6:**
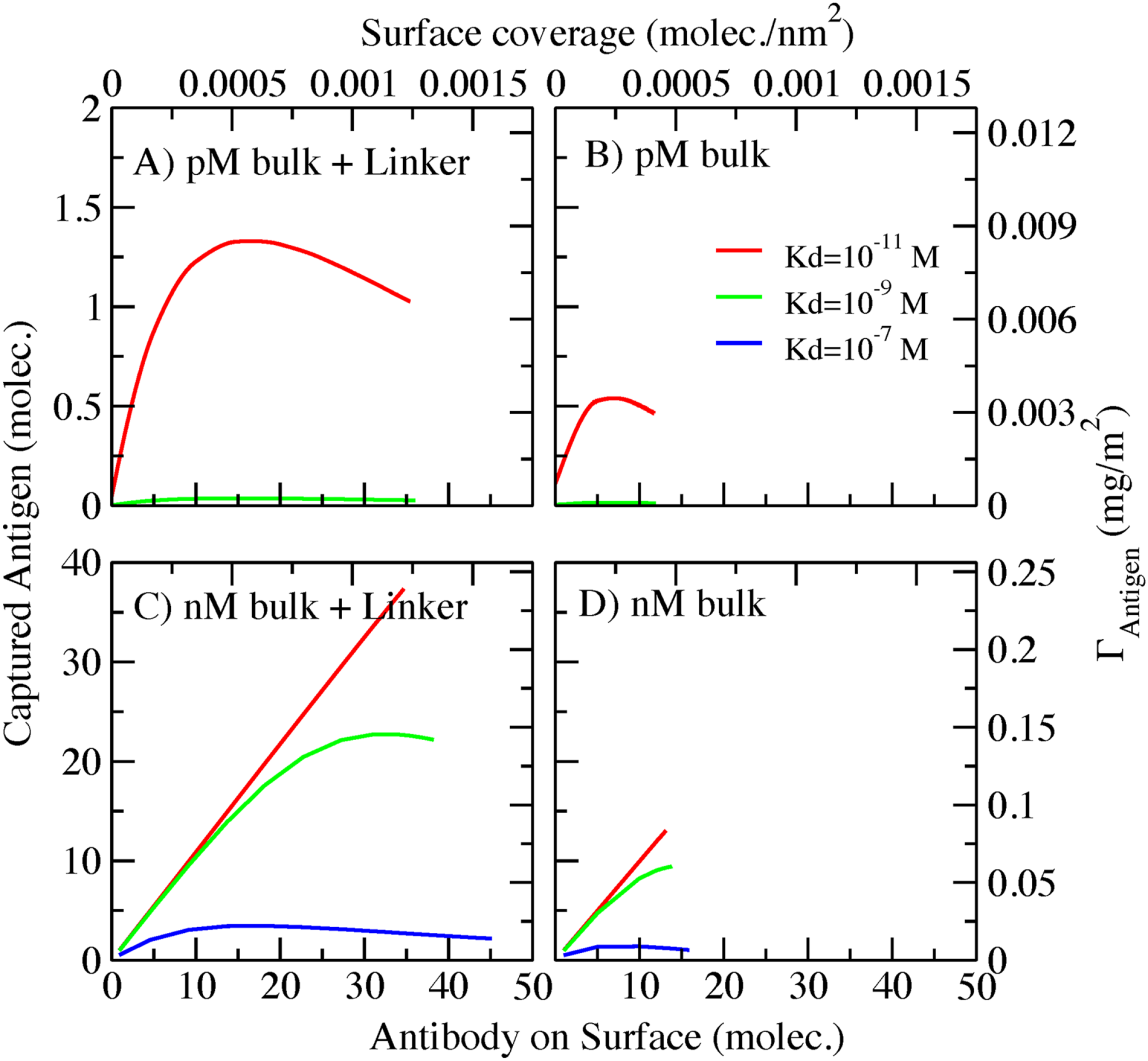
Captured antigen as a function of the antibody surface coverage on AcNP conjugated through streptavidin/biotin. The different colors represent different antibody-antigen Kd values. A) Bulk mix concentration = 20 pM antigen/10 pM antibody and spacer of 50 monomers. B) Bulk mix concentration = 20 pM antigen/10 pM antibody and no spacer. C) Bulk mix concentration = 200 nM antigen/100 nM antibody and spacer of 50 monomers. D) Bulk mix concentration = 200 nM antigen/100 nM antibody and no spacer.

Fig 6 shows the number of bound antibody-antigen pairs on the AcNP as a function of the number of bound antibodies on surface (biotinylated antibodies-streptavidin) for a bulk solution initially containing 10 pM antibody and 20 pM antigen (upper panels, Fig 6A and 6B) and a bulk mix initially containing 100 nM of antibody and 200 nM antigen (lower panels, Fig 6C and 6D), respectively. We have evaluated two spacer chain lengths: 50 monomers (left panels, Fig 6A and 6C) and no spacer (right panels, Fig 6B and 6D), and considered 3 different antibody-antigen dissociation constants (different color lines). Fig 6 illustrates the complexity of the binding on streptavidin-modified AcNPs. A general feature, similar to the behavior described for covalent conjugation (Fig 2), is the non-monotonic antigen-antibody binding on the NP surface. When the number of antibodies on the surface increases, the number of bound antigens displays a maximum, as a result of the entropic penalty of reorganization in a crowded surface.

Conjugation through streptavidin-biotin binding requires a completely different way to optimize the surface from that used with covalent conjugation (Fig 2). For a bulk solution prepared with low concentrations of the unbound species (10 pM of antibody and 20 pM of antigen; Fig 6A and 6B), we can observe that the amount of bound antigen is almost negligible. Under these conditions, even AcNPs having high affinity cannot bind considerable numbers of antigens (red line, Fig 6B). The addition of a spacer to the biotin-antibody system improves the amount of bound antibody-antigen on the streptavidin modified NP. This binding, however, is still on the order of a single bound pair, significantly lower than the binding achieved with the covalent conjugation architecture under similar conditions (Fig 2). This behavior is an important feature of this system that will condition the detection limit in LBAs. Antigen concentrations below picomolar levels can hardly be detected.

For a bulk solution prepared with relatively higher concentrations of the unbound species (200 nM of antigen and 100 nM of antibody; Fig 6C and 6D), binding depends almost linearly on antibody coverage when antigen affinity is high (Kd = 10^−11^ M). At weaker binding affinities, the capturing capability displays a maximum as antibody coverage increases. Note that both the system with and without a spacer shows such behavior. The main difference between these two situations is the enhancement and shift to higher surface coverage of the maxima for the system with the spacer. This is substantially different from the behavior observed in Fig 2 for the covalent modified AcNP where the addition of the spacer produces the non-monotonic behavior. Similarly to what occurs at low antigen concentration, the major difference between these two conjugation schemes is that the total number of captured antigens is much smaller using the streptavidin conjugation scheme (for high antigen concentration, compare Fig 6C and 6D with Fig 2C and 2D).

**Fig 7:**
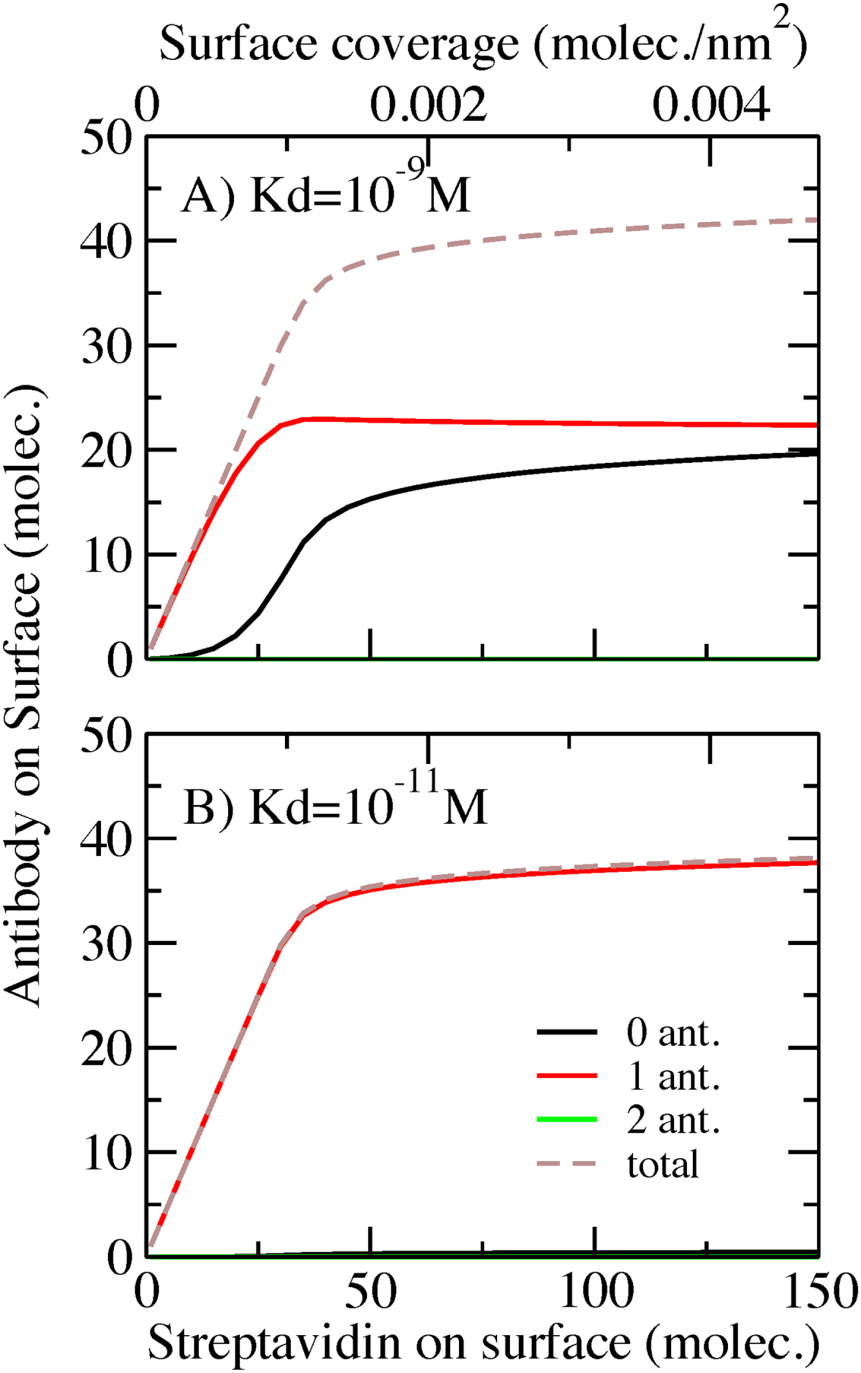
Streptavidin/biotin AcNP. Amount of antibody unbound (0 ant.), antibody bound to one antigen (1 ant.), antibody bound to two antigens (2 ant.) and total antibody (total) on surface as a function of the streptavidin surface coverage. A) Bulk mix concentration = 200 nM antigen/100 nM antibody, spacer 50 monomers, Kd = 10^−9^ M. B) Bulk mix concentration = 200 nM antigen/100 nM antibody, spacer 50 monomers, Kd = 10^−11^ M.

As previously mentioned, our theory provides molecular information about the species that are bound to the AcNP. In Fig 7, we show how the different antibody species (unbound, bound to one antigen, bound to two antigens) occupy the surface as a function of the number of streptavidin binding units for a low (Kd = 10^-9^M, panel A) and high Kd (Kd = 10^-11^M, panel B). These results correspond to antibodies bearing a spacer (50 monomers) and a bulk concentration resulting from mixing 200 nM of antigen with 100 nM of antibody.

In that figure (Fig 7), we observe the formation of a plateau at high streptavidin surface coverage, which corresponds to the limiting number of antibodies on surface observed in Fig 6. Adding more streptavidin in this plateau region does not increase the number of antibodies on surface. Due to crowding effects, an important number of the antibodies on surface are unbound at low Kd (black line in Fig 7A). The dominant species, however, is antibodies bound to one antigen. Under these conditions, antibodies with two antigens are not present on the surface, due to high volume that they occupy. Namely, the small decrease in binding free energy is not sufficient to counterbalance the large entropic cost of surface reorganization upon capturing two antigens. When the strength of the antibody-antigen interaction increases (Fig 7B Kd = 10^-11^M) antibodies with one antigen are the only species on the surface. This result explains why in Fig 6 we observe a linear binding behavior with antibody coverage for Kd = 10^-11^M.

Interestingly, stronger antibody-antigen affinity results in slightly fewer antibodies on the surface (compare dashed lines curves for both panels of Fig 7). However, antigen-capture increases with affinity (see also Fig 6C). This is a clear example of how the surface, due to the presence of the streptavidin-biotin chemical equilibrium, can optimize the surface coverage to increase the free energy gain associated with the binding equilibriums and reduce the entropic cost arising from crowding.

If we consider the average position of the center of mass of the antibodies in the streptavidin AcNP (see S1 Text), the results show less complexity than those of covalently conjugated antibodies. The possibility of self-regulating antibody coverage when using streptavidin conjugation implies that increasing surface coverage does not have a high impact on the possible conformations. Then, the average center of mass position remains approximately the same when increasing streptavidin coverage for the different antibody species. Antibodies with two antigens have the most extended conformations. However, this bulky molecule is nearly absent from the surface (see Fig 7). Unbound antibodies remain the least extended conformation, while the conformations of antibodies with one antigen are slightly extended to account for the antigen volume.

**Fig 8:**
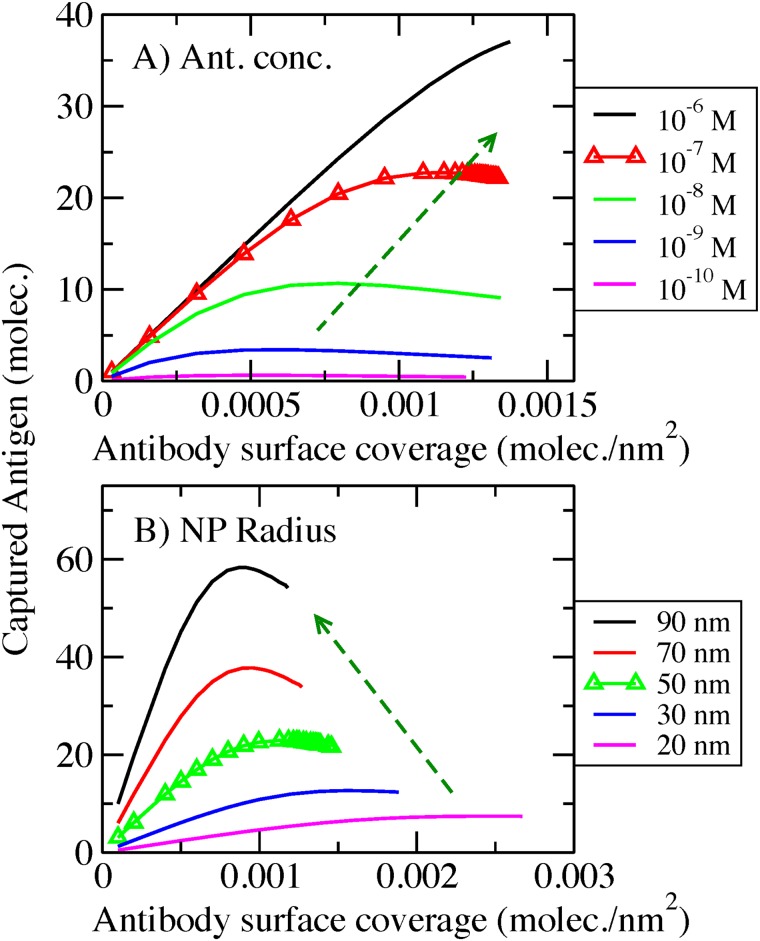
Captured antigen per unit area as a function of antibody surface coverage. Streptavidin conjugated AcNP, spacer 50 monomers, Kd = 10^−9^ M. A) For different antigen bulk concentrations. AcNP radius = 50 nm. B) For different AcNP radius. Inset in panel B) are same data but the captured antigen normalized by the area of the NP. Red and black dashed lines, in the inset, represent the ideal situation of all the antibodies bound to two or one antigen respectively. Bulk concentration mix = 100 nM antibody: 200nM antigen. Triangle symbols represent the set of conditions used in this paper for the rest of the calculations.

Next we analyze the impact of antigen/antibody bulk concentration and nanoparticle radius on the number of bound antibody-antigen pairs on the AcNP. Fig 8 shows the amount of captured antigen as function of streptavidin coverage, for an AcNP with antibodies having a 50-monomer spacer and Kd = 10^-9^M, at different bulk concentrations (Fig 8A) and different AcNP radius (Fig 8B). In Fig 8A, we see that increasing the concentration of the bulk species enhances antibody-antigen binding. Antigen capture displays a roughly linear behavior as function of the surface coverage. This is particularly different from what we observe in the system with covalently bonded antibodies, where increasing bulk concentration leads to a more pronounced non-monotonic behavior (Fig 5A), which results from balancing ligand-receptor binding and the conformational constraints of accommodating bulky molecules. The surface of the streptavidin AcNP can reorganize and regulate the number of adsorbed antibodies as a result of the additional ligand-receptor equilibrium. Then, as we increase the bulk concentration binding rises but the non-monotonic behavior is less pronounced. Note that the maximum amount of antibody on the surface (end-point of the binding curves) is approximately the same independently of bulk concentration. This suggests that despite the increment in the chemical potential of the solution species, the surface can only accommodate so many antibodies.

Now we consider the impact of nanoparticle size on the number of bound antibody-antigen pairs. In the covalently modified system, the radius of the AcNP has a large impact in the binding behavior, with a vanishing non-monotonic behavior for small radius (Fig 5B). A similar feature is observed in the streptavidin conjugated AcNP, as seen in Fig 8B that shows antigen binding for different nanoparticle radius. This dependence on size is due to the effect that curvature has on the available volume to accommodate bulky molecules near the surface. A high curvature (small radius) results in more available volume for the spacer-antibody to sample conformational space and bind at different distances from the surface, which reduces the criticality in the balance between binding and crowding, thus diminishing the non-monotonical behavior. Fig 8B also shows that the number of bound antibody-antigen pairs is higher for larger AcNP due to the larger surface area.

In Fig 8B we also included an inset with the same data but the number of captured antigen had been normalized by the area of the nanoparticle. This inset provide information about the efficiency of binding. Red and black dashed lines in the inset represent the ideal situation of all the antibodies bound to two or one antigen respectively. We can observe that AcNP with smaller diameters tend to be more efficient in capturing antigen. As we mentioned before, this is a consequence of the increment in curvature (smaller diameters) that provides more available volume and then less entropic penalty to the binding. Is interesting to note that the difference with Fig 5B inset is that in this system (Fig 8B inset) the binding barely exceeds the ideal behavior of one antigen per antibody (black dashed line). This behavior is again a manifestation of the extra degree of freedom that the streptavidin/biotin bond provides to compensate for the entropic penalty due to crowding.

## Discussion and conclusion

In this work we have presented a set of results to shed light into the behavioral complexity of the ligand-receptor binding of voluminous ligands in crowded surfaces. In particular, we have analyzed the case of antibody conjugated nanoparticles (AcNP) since antibodies are large ligands of utmost importance in biomedical applications. We have mainly focused on understanding the non-monotonic behavior observed in the ligand-receptor pair formation as a function of surface coverage, which has been reported in several experimental works [15–20]. For this purpose, we used a molecular theory that has been successfully applied to study similar systems [22–26] showing good agreement with experimental results. Using this approach, we have explored two different systems: an AcNP with antibodies covalently bonded to its surface, and an AcNP with antibodies bound using the highly specific streptavidin-biotin interaction. Our calculations have explored the binding behavior of these systems varying the bulk solution concentration, antibody-antigen affinity, the inclusion of a polymer spacer, and the radius of the nanoparticle. The main result we report is a very complex ligand-receptor binding behavior, which strongly depends on the competition between binding and molecular reorganization on the surface due to the crowding effect introduced by the volume occupied by the large ligand (antibody) and ligand-receptor pair (antibody-antigen).

For AcNPs conjugated through covalent bonds with no spacer, our calculations show that binding has a linear behavior at low surface coverage to then reach a plateau at high coverage. This behavior is similar observations of Saha et al. [19] in their experimental work. An important difference is that in those experiments the antibodies were immobilized at random orientations while in our calculation antibodies are anchored to the surface through the Fc-domain. Besides these differences in architecture, both systems display similar behavioral features (Fig 2D and Fig 2 from [19]), suggesting that crowding of the surface is one of the major factors affecting AcNP binding. The inclusion of a spacer greatly improves the amount of bound ligand-receptor pairs. At the same time, inclusion of a spacer results in a more pronounced non-monotonic behavior (maxima in Fig 2). These results are consistent with the findings of Brogan et al. [16] showing that the addition of a spacer on a Fab-fragment conjugated NP increases the amount of bound pairs. In addition, Brogan et al. showed that there is a maximum in the capturing capability as a function of the surface coverage of Fab-fragments.

Regarding solution conditions, increasing antigen bulk concentration significantly shifts the position of maximum binding to higher surface coverage. For AcNP and LBA design, this implies that the number of large ligands that decorate the surface must be appropriately chosen depending on bulk receptor concentration. Signal detection for *in-vitro* applications will have high variability depending on environment composition. Regarding size, small AcNPs give a linear behavior that eliminates the uncertainties associated with finding the optimal conditions for binding. However, the total amount of bound pairs is substantially higher in larger AcNPs. Thus, given the maximum NP size associated with particular application constraints, the design of covalently modified AcNP with optimal binding properties will likely require dealing with the complications of non-monotonic behavior.

Our theory provides information about molecular organization. Although antibodies cannot detach from the covalently modified architecture, the AcNP can reorganize the surface optimizing the number of bound antigens and the configuration of the spacer-antibody. We predict that antibodies with two antigens, due to a volume constraint, are only possible at very low surface coverage. Antibodies with a single bound antigen represent the most important contribution to the total number on the surface. In the plateau region, increasing the surface coverage results in the mere addition of unbound antibodies. The maximum in the binding capability occurs at the transition between collapsed and stretched conformations of the spacer.

AcNPs with streptavidin-biotin conjugation present very different behavior from their covalent counterpart. Streptavidin AcNPs are significantly less sensitive at picomolar concentrations. The binding behavior of these AcNPs with streptavidin is qualitatively similar with and without a spacer, although the addition of a spacer substantially increases binding and shifts the curves to higher surface coverage. We predict that increasing the antibody-antigen affinity diminishes the non-monotonical behavior, yielding a linear response for the lowest studied Kd (10^-11^M). An interesting feature in the behavior of AcNPs conjugated through streptavidin is the presence of a maximum in the number of antibodies that the nanoparticle can accommodate on its surface. After this maximum, increasing the number of streptavidin binding sites does not modify the number of biotinylated antibodies on the surface. This result is a clear manifestation of the high entropic cost associated with surface crowding. At high surface coverage, the entropy cost of crowding prevails over the free energy decrease associate with streptavidin-biotin binding. In other words, at high surface coverage and despite the high biotin-streptavidin affinity, the system chooses to have some unbound streptavidin on the surface in order not to pay the price of further disrupting the crowded environment, which would occur upon bringing additional bulky molecules (biotinylated antibody) to the surface. This behavior has been observed in related systems [15, 36].

Surface reorganization in the AcNP means finding the optimal conditions that yield thermodynamic equilibrium, which results from the interplay between the free energy decrease of binding and the effects of molecular crowding. In the covalently conjugated AcNP, the surface reorganizes by including different species and by collapsing or stretching the spacer-antibody units. In the streptavidin AcNP, the surface reorganizes by including different species and by regulating the number of antibodies on the surface. A transition between collapsed-elongated configurations does not occur in this latter system. For high antibody-antigen affinities (Kd = 10^-11^M), every antibody adsorbed in the streptavidin-AcNP bears a single antigen.

We have also examined the binding behavior as a function of the (bulk) solution concentrations and the radius of the AcNPs. In contrast with the covalently conjugated AcNP, the non-monotonic behavior of a streptavidin AcNPs is less pronounced as bulk concentrations are increased. Interestingly, the maximum number on antibodies that can be placed on the surface is approximately the same independently of the bulk concentration. We predict that the non-monotonic binding behavior diminishes and eventually vanishes as AcNP radius decreases. A higher curvature (smaller radius) offers a larger available volume for sampling spacer configurations, thus allowing for binding at different distances from the NP surface. However, for sensing purposes, larger AcNPs are more relevant because they can capture significantly more antigen. As a compromise, these large particles display more pronounced non-monotonic binding behavior and can make the design of experiments more complicated.

In conclusion, we have presented a series of theoretical predictions that can be useful in the future design of antibody-conjugated nanoparticles for biomedical and sensing applications. We have addressed the behavior resulting from two different conjugation schemes and pointed out critical differences between them. Our results have been presented in terms of normalized quantities to facilitate extrapolation to other similar systems. One of the noteworthy features of AcNPs is the non-monotonic binding behavior (maximum) resulting from the competition between the antigen-antibody (and streptavidin-biotin) binding and the entropic costs of surface reorganization to prevent steric repulsions under the crowded nano-environment that these large glycoproteins impose.

## Supporting information

S1 Text In this file we included the necessary equations and molecular details of the molecular theory. (DOCX)
