# Supplementary material for: Behavior of ligand binding assays with crowded surfaces: Molecular model of antigen capture by antibody-conjugated nanoparticles
Source: PLoS One. 2017 Sep 28;12(9):e0185518. doi: 10.1371/journal.pone.0185518 (PMC5619776; doi:10.1371/journal.pone.0185518)
Supplement: S1 Text — (DOCX) [file pone.0185518.s001.docx]

**Supporting Information (S1 Text):**

**Coarse-grained model:**

The molecular model of the antibody conformations is a coarse grained model based on the crystallographic data of an anti-HIV-1 b12 monoclonal antibody [1]. The coarse grain model of the complex antibody-antigen was build by combining the crystallographic data of b12 antibody and the complex b12-Fab/HIV-gp120 [2] obtained from the protein data bank (PDB) structures 1HZH and 2NY7. The dissociation constant range is based on the experimental work of Landry et al [8] that suggest a Kd range of 10^-8^ to 10^-11^ M for different antibody/antigens. The coarse grain is such that each amino acid in the structure is represented by a bead of 6 nm diameter, centered in the alpha carbon of that residue. The spacer conformation is based on a coarse grain model of poly-ethylene-glycol (PEG) and each bead (diameter=6 nm) represents one monomeric unit. The antibody structure is fix in its initial conformation obtained from the PDB, but with full 3D rotational and translational freedom. In the case of the spacer we also include conformational changes. The spacer conformations were generated by choosing randomly between the possible orientations of each monomer in the chain (trans, gauche + and gauche -)[3, 4]. An additional 10% of chains biased toward stretched conformations were added to account for the more stretched conformation observed in polymer brushes. In the case of the streptavidin modified AcNP, to simplify the calculations, the streptavidin is considered as a punctual binding site on the surface with no volume.

**Molecular Theory:**

To study the binding between antigen molecules and an antibody-conjugated nanoparticle (AcNP), we have used a molecular theory that computes the minimum free energy of this system[5, 6]. In this section, we describe this theoretical approach for the two conjugation strategies considered in the present work: covalent conjugation and streptavidin-biotin conjugation. In order to facilitate the description of the theoretical framework, we will describe the systems in the inverse order to the one presented in the article.

**1) AcNP with streptavidin-biotin conjugation:**

The nanoparticle (NP) is described as spherical surface of radius $R_{NP}$ modified with streptavidin molecules, such that there are $N_{S}$ free sites $(S)$ available for biotin binding. The NP is immersed in a solution at constant temperature $T$ that contains water ($w$), antigens ($a$), and biotinylated antibodies that can be either antigen-free ($A$) or form complexes with one ($Aa$) or two antigens ($Aaa$). These biotinylated antibodies can also include a small polymeric spacer that increases the conformational possibilities of the antibody.

**1.a) Free energy:**

Assuming spherical symmetry, the total Helmholtz free energy of this system (F), using the molecular theory, can be expressed as:

$$\beta F=4\pi\int_{R_{NP}}^{\infty} {dr r}^{2} \rho_{w}\left( r \right)\left[ \ln\rho_{w}\left( r \right)v_{w}-1 \right]$$

$$+\sum_{j\in\left\{ a,A,Aa,Aaa \right\}} \left( 4\pi\int_{R_{NP}}^{\infty} {dr r}^{2} \sum_{\alpha_{j}^{sol}} \rho_{j}\left( \alpha_{j}^{sol},r \right)\left[ \ln\rho_{j}\left( \alpha_{j}^{sol},r \right)v_{w}-1+\beta\mu_{j}^{⊖} \right] \right)+N_{S}\sum_{i\in\left\{ A,Aa,Aaa \right\}} f_{i}^{NP}\left( \sum_{\alpha_{i}^{NP}} P_{i}\left( \alpha_{i}^{NP} \right)\ln P_{i}\left( \alpha_{i}^{NP} \right) \right)$$

${+ N}_{S}\sum_{k\in\left\{ S,A,Aa,Aaa \right\}} f_{k}^{NP}\left( \ln f_{k}^{NP}+\beta\mu_{k}^{NP⊖} \right)$ Eq. (1)

Where $r$ gives the distance with respect to the center of the nanoparticle, and $\beta=\frac{1}{k_{B}T}$ where $k_{B}$ is the Boltzmann constant.

The first term in this expression of the free energy is the translational entropy of the solvent (water); where $\rho_{w}\left( r \right)$ is the local molar density of water at $r$ and $v_{w}$ its molecular volume.

The second term is the sum over mobile species in the solution (except water) that includes translational and configuracional entropy of these molecules as well as their self-energies, where $\mu_{j}^{⊖}$with represent standard chemical potential of the specie $j\in\left\{ a,A,Aa,Aaa \right\}$. In this free energy term, $\rho_{j}\left( \alpha_{j}^{sol},r \right)$ is the molar density at $r$ of species $j$ in its molecular conformation in solution, $\alpha_{j}^{sol}$. These $\alpha$ conformations include different molecular rotations, and in the case of spacer-modified antibodies (free or antigen-complexed) they also describe different conformers of the flexible polymer. We use superscripts “sol” and “NP” to differentiate between conformations of molecules in the solution and bound to the nanoparticle, respectively.

The third term that contributes to $F$ is the conformational entropy of species that are bound to the surface, where $P_{i}\left( \alpha_{i}^{NP} \right)$ is the probability of a conformation $\alpha_{i}^{NP}$of species $i\in\left\{ A,Aa,Aaa \right\}$.

The last term that contributes to $F$ is the mixing entropy of species bound to the nanoparticle surface, and the self-energy of these molecules, where $\mu_{k}^{NP⊖}$ is the standard chemical potential of species $k\in\left\{ S,A,Aa,Aaa \right\}$.

**1.b) Bead density:**

Thus far, our theoretical approach has been described in terms of molecular densities. But in order to incorporate molecular details of the antibody, antigen, and complexes we need to translate these densities into coarse-grained bead densities. The bead density of j is expressed as$n_{j}\left( \alpha_{j};r,r' \right)$ with $j\in\left\{ a,A,Aa,Aaa \right\}$ . This bead density is the number of coarse grain beads of a specie j at a distance $r'$ that correspond to a conformation $\alpha_{j}$, this conformation is located at distance $r$ from the center of the NP.

The total density of solution species $j\in\left\{ a,A,Aa,Aaa \right\}$ can be expressed as:

$\left\langle\rho_{j}\left( r \right) \right\rangle=\sum_{\alpha_{j}^{sol}} \rho_{j}\left( \alpha_{j}^{sol},r \right)$ Eq. (2)

Where angle brackets denote that this sum can be interpreted as an ensemble average. Similarly, the total density of molecular coarse-grained beads of species $j$ at $r$can be written as:

$\left\langle n_{j}^{sol}\left( r \right) \right\rangle=4\pi\sum_{\alpha_{j}^{sol}} \int_{R_{NP}}^{\infty} {dr' r'}^{2} \rho_{j}\left( \alpha_{j}^{sol},r' \right) n_{j}\left( \alpha_{j}^{sol};r^{'},r \right)$ Eq. (3)

Where $n_{j}\left( \alpha_{j}^{sol};r^{'},r \right)$ is the number of beads that a molecule positioned at $r'$ in configuration $\alpha_{j}^{sol}$ contributes to $r$. Note that in Eq. (3) we have assumed that all molecular beads of the species have the same volume. In a similar way, the number of beads that a molecule bound to the surface contributes to at distance $r$ from the center of the nanoparticle is:

$\left\langle n_{i}^{NP}\left( r \right) \right\rangle=\sum_{\alpha_{i}^{NP}} P_{i}\left( \alpha_{i}^{NP} \right)n_{i}\left( \alpha_{i}^{NP};R_{NP},r \right)$. Eq. (4)

This beads distribution, $n_{j}\left( \alpha_{j}^{sol};r^{'},r \right)$, is an input of our theoretical approach that must be provided for all conformations and positions by a molecular model.

**1.c) Constraints:**

Three physical and chemical constraints must be imposed to the free energy that we have written. First, the fluid is incompressible. This condition implies that every element of volume is completely occupied by some of the molecular species, which can be expressed as:

$\rho_{w}\left( r \right)v_{w}+\sum_{j\in\left\{ a,A,Aa,Aaa \right\}} \left\langle n_{j}^{sol}\left( r \right) \right\rangle v_{j}+N_{s}\sum_{i\in\left\{ A,Aa,Aaa \right\}} f_{i}^{NP}\left\langle n_{i}^{NP}\left( r \right) \right\rangle v_{i}=1$ Eq. (5)

Where the fraction of streptavidin sites occupied by molecules of type $i$ with $i\in\left\{ A,Aa,Aaa \right\}$ is $f_{i}^{NP}$. Thus, the number of one-antigen antibodies on the nanoparticle is $N_{S}f_{Aa}^{NP}$, and similarly for $A$ and $Aaa$. The fraction of free streptavidin sites is $f_{S}^{NP}$, such that:

$f_{S}^{NP}+f_{A}^{NP}+f_{Aa}^{NP}+f_{Aaa}^{NP}=1$ Eq. (6)

In addition, the total number of antibody ($N_{A}$) and antigen ($N_{a}$) molecules incorporated into the solution must be conserved regardless of complexes that they may form. These two constraints can be written in the following way:

$N_{A}=N_{S}\left( 1-f_{S}^{NP} \right)+\sum_{i\in\left\{ A,Aa,Aaa \right\}} 4\pi\int_{R_{NP}}^{\infty} {dr r}^{2} \sum_{\alpha_{i}^{sol}} \rho_{i}\left( \alpha_{i}^{sol},r \right)$ Eq. (7)

$N_{a}=N_{S}\left( f_{Aa}^{NP}+f_{Aaa}^{NP} \right)+\sum_{j\in\left\{ a,Aa,Aaa \right\}} 4\pi\int_{R_{NP}}^{\infty} {dr r}^{2} \sum_{\alpha_{j}^{sol}} \rho_{j}\left( \alpha_{j}^{sol},r \right)$ Eq. (8)

The proper thermodynamic potential that describes equilibrium under the experimental conditions that we are considering is the semi-grand potential $\Omega$, which is the Laplace transform of $F$ having the chemical potentials of mobile species constant. Due to the three aforementioned constraints only two (out of five) chemical potentials are independent. Thus:

$\Omega\left( T,V,\mu_{w},\mu_{a},\mu_{A},\mu_{Aa},\mu_{Aaa} \right)\equiv F-\mu_{A}N_{A}-\mu_{a}N_{a}-\mu_{Aa}N_{Aa}-\mu_{Aaa}N_{Aaa}$ Eq. (9)

**1.d) Optimization:**

Functional optimization of the semi-grand potential with respect to all explicit functions, considering the incompressibility constraint, leads to expressions for each of these quantities. For the density of water such procedure yields to:

$\rho_{w}\left( r \right)v_{w}=\exp\left( -\beta\pi\left( r \right)v_{w} \right)$ Eq. (10)

Where the osmotic pressure potential, $\pi\left( r \right)$, is the local Lagrange multiplier introduced to reinforce fluid incompressibility.

For the density of the other mobile species, we obtain:

$\rho_{j}\left( \alpha_{j}^{sol},r \right)v_{w}=\exp\left( \beta\mu_{j}-\beta\mu_{j}^{⊖}-4\pi\int_{R_{NP}}^{\infty} {dr' r'}^{2} n_{j}\left( \alpha_{j}^{sol};r,r^{'} \right)\beta\pi\left( r^{'} \right)v_{j} \right)$ Eq. (11)

With $j\in\left\{ a,A,Aa,Aaa \right\}$, where the chemical potentials satisfy the following equations, which guaranty chemical equilibrium between antibodies, antigens and their complexes:

$\mu_{Aa}=\mu_{A}+\mu_{a}$ Eq. (12)

$\mu_{Aaa}=\mu_{A}+{2\mu}_{a}$ Eq. (13)

Optimization of $\Omega$ with respect to the probability distribution of conformations of the nanoparticle-bound species leads to:

$P_{i}\left( \alpha_{i}^{NP} \right)=\frac{1}{Q_{i}}\exp\left( -4\pi\int_{R_{NP}}^{\infty} {dr' r'}^{2} n_{i}\left( \alpha_{i}^{NP};R_{NP},r' \right)\beta\pi\left( r^{'} \right)v_{i} \right)$ Eq. (14)

With $i\in\left\{ A,Aa,Aaa \right\}$, where $Q_{i}$ is the partition function introduced to ensure $\sum_{\alpha_{i}^{NP}} P_{i}\left( \alpha_{i}^{NP} \right)=1$.

The fraction of streptavidin sites occupied by an antibody species, can be obtained from:

$\frac{f_{i}^{NP}}{f_{S}^{NP}}=Q_{i}\exp\left( \beta\mu_{i}-\beta\mu_{i}^{NP⊖}+\beta\mu_{S}^{NP⊖} \right)$ Eq. (15)

At this point, all explicit functions that compose the free energy only depend on one interaction potential besides input variables, the local osmotic pressure, $\pi\left( r \right)$. This position-dependent potential can be numerically calculated discretizing space and using the local incompressibility of the fluid Eq. (5), which provides a set of coupled non-linear differential equations. One we have obtained the osmotic pressure at all distances from the nanoparticle, all free energy terms can be calculated. Thus, any thermodynamic observable of interest can be derived from the free energy. Structural properties can be calculated as ensemble average over conformations using the corresponding probability distributions.

**1.e) Equilibrium constants:**

The next is a summary of the equilibrium constants used to solve the molecular theory for the AcNP system.

$K_{a,j}^{0}$ is the association equilibrium constant for the formation of the complex j. $K_{a,j}^{0}$ allows to determine standard chemical potentials from the following formula:

$K_{a,j}^{0}=\exp\left( \beta\mu_{FS}^{NP⊖}+\beta\mu_{i}^{⊖}-\beta\mu_{i}^{NP⊖} \right)$ $j\in\left\{ A,Aa,Aaa \right\}$ Eq. (16)

The following are the association constants for used in this work:

$K_{a,streptavidin-biotin}^{0}$= 1*10^14^ [M] [7]

$K_{a,Antibody-antigen}^{0}$= variable (1*10^7^ [M],1*10^9^ [M],1*10^11^ [M]) [8]

$K_{a,Antibody-antigen-antigen}^{0}$= 15* $K_{a,Aa}^{0}$ (based on the avidity ratio observed in b12 antibodies[9])

**2) AcNP covalent conjugation:**

The theoretical treatment of the molecular theory for the AcNP with covalent conjugation is a simplification of our previous discussed system. Instead of having the possibility of having antibodies in solution, now all the antibodies remain attached to the surface of the NP through a covalent bond. The following equations are derived for this system. Due to the similarity with the previous system we reduce the description of the equations.

**2.a) Free energy:**

The Helmholtz free energy is the following:

$$\beta F=4\pi\int_{R_{NP}}^{\infty} {dr r}^{2} \rho_{w}\left( r \right)\left[ \ln\rho_{w}\left( r \right)v_{w}-1 \right]+4\pi\int_{R_{NP}}^{\infty} {dr r}^{2} \sum_{\alpha_{a}^{sol}} \rho_{a}\left( \alpha_{a}^{sol},r \right)\left[ \ln\rho_{a}\left( \alpha_{a}^{sol},r \right)v_{w}-1+\beta\mu_{a}^{⊖} \right]+N_{A}\sum_{i\in\left\{ A,Aa,Aaa \right\}} f_{i}^{NP}\left( \sum_{\alpha_{i}^{NP}} P_{i}\left( \alpha_{i}^{NP} \right)\ln P_{i}\left( \alpha_{i}^{NP} \right) \right)$$

$+N_{A}\sum_{i\in\left\{ A,Aa,Aaa \right\}} f_{i}^{NP}\left( \ln f_{i}^{NP}+\beta\mu_{i}^{NP⊖} \right)$ Eq.(17)

$N_{A}$ is the total number of antibodies covalently grafted to the surface

**2.b) Bead density:**

In the solution we only have the solvent and the antigen.

$\left\langle\rho_{a}\left( r \right) \right\rangle=\sum_{\alpha_{a}^{sol}} \rho_{a}\left( \alpha_{a}^{sol},r \right)$ Eq.(18)

$\left\langle n_{a}^{sol}\left( r \right) \right\rangle=4\pi\sum_{\alpha_{a}^{sol}} \int_{R_{NP}}^{\infty} {dr' r'}^{2} \rho_{a}\left( \alpha_{a}^{sol},r' \right) n_{a}\left( \alpha_{a}^{sol};r^{'},r \right)$ Eq.(19)

On the other hand, on the surface of the NP we can have all the species:

$\left\langle n_{i}^{NP}\left( r \right) \right\rangle=\sum_{\alpha_{i}^{NP}} P_{i}\left( \alpha_{i}^{NP} \right)n_{i}\left( \alpha_{i}^{NP};R_{NP},r \right)$ $i\in\left\{ A,Aa,Aaa \right\}$ Eq.(20)

Notice that $R_{NP}$ in these expressions is redundant. We keep it for consistency.

**2.c) Constraints:**

The incompressibility constraint leads to:

$f_{A}^{NP}+f_{Aa}^{NP}+f_{Aaa}^{NP}=1$ Eq.(21)

$\rho_{w}\left( r \right)v_{w}+\left\langle n_{a}^{sol}\left( r \right) \right\rangle v_{a}+N_{A}\sum_{i\in\left\{ A,Aa,Aaa \right\}} f_{i}^{NP}\left\langle n_{i}^{NP}\left( r \right) \right\rangle v_{i}=1$ Eq.(22)

NA is the total number of antibodies grafted to the surface, including antigen-complexed molecules

And the mass conservation for the antigen in solution is:

$N_{a}=N_{A}\left( f_{Aa}^{NP}+f_{Aaa}^{NP} \right)+4\pi\int_{R_{NP}}^{\infty} {dr r}^{2} \sum_{\alpha_{a}^{sol}} \rho_{a}\left( \alpha_{a}^{sol},r \right)$ Eq.(23)

The semi-grand canonical potential is:

$\Omega\left( T,V,\mu_{w},\mu_{a} \right)\equiv\Omega\left( T,V,\mu_{a} \right)=F-\mu_{a}N_{a}$ Eq.(24)

**2.d) Optimization:**

When we do the optimization using the Lagrange multiplier method, we obtain the following equations:

$\rho_{w}\left( r \right)v_{w}=\exp\left( -\beta\pi\left( r \right)v_{w} \right)$ Eq.(25)

$\rho_{a}\left( \alpha_{a}^{sol},r \right)v_{w}=\exp\left( \beta\mu_{a}-\beta\mu_{a}^{⊖}-4\pi\int_{R_{NP}}^{\infty} {dr' r'}^{2} n_{a}\left( \alpha_{a}^{sol};r,r^{'} \right)\beta\pi\left( r^{'} \right)v_{a} \right)$ Eq.26

$P_{i}\left( \alpha_{i}^{NP} \right)=\frac{1}{Q_{i}}\exp\left( -4\pi\int_{R_{NP}}^{\infty} {dr' r'}^{2} n_{i}\left( \alpha_{i}^{NP};R_{NP},r' \right)\beta\pi\left( r^{'} \right)v_{i} \right)$ Eq.(27)

$\frac{f_{i}^{NP}}{f_{A}^{NP}}=Q_{i}\exp\left( \beta\mu_{a}-\beta\mu_{i}^{NP⊖}+\beta\mu_{A}^{NP⊖} \right)$ Eq.(28)

$$i\in\left\{ Aa,Aaa \right\}$$

**2.e) Equilibrium constants:**

Finally the equilibrium constants are the same used in the previous system, but without the equilibrium streptavidin/biotin:

$K_{a}^{0}=\exp\left( \beta\mu_{Aa}^{NP⊖}-\beta\mu_{A}^{NP⊖}-\beta\mu_{a}^{⊖} \right)$ Eq.(29)

$K_{a}^{0}=\exp\left( \beta\mu_{Aaa}^{NP⊖}-\beta\mu_{Aa}^{NP⊖}-\beta\mu_{a}^{⊖} \right)$ Eq.(30)

$K_{a,Aa}^{0}$= variable (1*10^7^ [M],1*10^9^ [M],1*10^11^ [M])[8]

$K_{a,Aaa}^{0}$= 15* $K_{a,Aa}^{0}$ (based on the avidity ratio observed in b12 antibodies[9])

**References:**

1. Saphire, E.O., et al., Crystal structure of a neutralizing human IGG against HIV-1: a template for vaccine design. Science, 2001. 293(5532): p. 1155-9.

2. Zhou, T., et al., Structural definition of a conserved neutralization epitope on HIV-1 gp120. Nature, 2007. 445(7129): p. 732-7.

3. Flory, P.J., Statistical mechanics of chain molecules, ed. Interscience. 1969, New York.

4. Longo, G. and I. Szleifer, Ligand-receptor interactions in tethered polymer layers. Langmuir, 2005. 21(24): p. 11342-51.

5. M. A. Carignano, I.S., Statistical thermodynamic theory of grafted polymeric layers. J Chem Phys, 1993. 98(6).

6. Szleifer, I., Protein Adsorption on Surfaces with Grafted Polymers: A Theoretical Approach. Biophysical Journal, 1997. 72(2 Pt 1): p. 595-612.

7. Ren, C.L., et al., Streptavidin-biotin binding in the presence of a polymer spacer. A theoretical description. Langmuir, 2009. 25(20): p. 12283-92.

8. Landry, J.P., Y.Y. Fei, and X.D. Zhu, Simultaneous Measurement of 10,000 Protein-Ligand Affinity Constants Using Microarray-Based Kinetic Constant Assays. Assay and Drug Development Technologies, 2012. 10(3): p. 250-259.

9. Klein, J.S., et al., Examination of the contributions of size and avidity to the neutralization mechanisms of the anti-HIV antibodies b12 and 4E10. Proc Natl Acad Sci U S A, 2009. 106(18): p. 7385-90.
